# Packet Key-Based End-to-End Security Management on a Blockchain Control Plane

**DOI:** 10.3390/s19102310

**Published:** 2019-05-19

**Authors:** Younchan Jung, Marnel Peradilla, Ronnel Agulto

**Affiliations:** 1 School of Information, Communications and Electronics Engineering, The Catholic University of Korea, 43 Jibong-ro, Bucheon-si, Gyeonggi-do 14662, Korea; ronnelagulto@catholic.ac.kr; 2 College of Computer Studies, De La Salle University, 2401 Taft Avenue, Manila 1004, Philippines; marnel.peradilla@dlsu.edu.ph

**Keywords:** blockchain-based security control, security management, vertical model, SDN-based horizontal model, packet key

## Abstract

The existing LTE mobile system uses the vertical model to handle the session-based security management. However, the goal of this paper is to propose a packet key-based security management scheme on the blockchain control plane to enhance the existing session key-based security scheme and overcome the limitation that the existing vertical model, as well as the Software-Defined Networking (SDN) based horizontal model, confronts within solving end-to-end security management. The proposed blockchain-based security management (BSM) scheme enables each peer to easily obtain the necessary parameters required to manage the packet key-based security system. The important features of the BSM scheme include the renewal process, which enables the different packet data streams to use completely different security parameters for the security management. In addition, because even blind values cannot be exposed to the possible attackers, our BSM scheme guarantees very secure end-to-end data transfer against active attacks such as falsification of data and transactions. Finally, this paper compares the BSM scheme with the existing vertical model to prove the advantageous effects on latency.

## 1. Introduction

**Figure 1:**
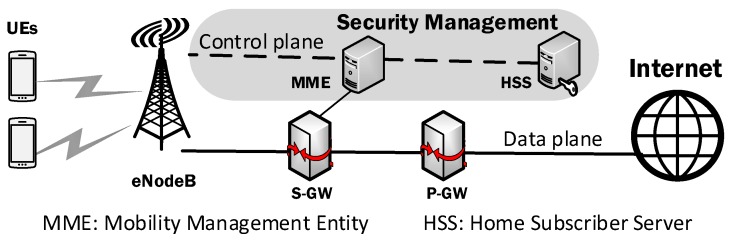
Security management based on the vertical model distributed servers such as Mobility Management Entity (MME) and Home Subscriber Server (HSS) in LTE networks.

The main feature of the Long Term Evolution (LTE) system is to use two independent planes where the signaling information process is done by control plane and the user data process is done by a user data plane [1,2,3,4,5]. As depicted in Figure 1, security management functions are based on the vertical model which uses distributed servers such as Mobility Management Entities (MMEs) and Home Subscriber Servers (HSSs) in LTE networks. Thus, the integrated operation of network control on the control plane enables security management functions to be efficiently implemented within the range of local LTE networks. However, for the end-to-end security management throughout the Internet, different solutions have been explored to use the horizontal network function model [6,7,8,9].

The existing Session Initiation Protocol (SIP) based Voice over Internet Protocol (VoIP) call system uses the vertical model to handle the security management where multiple servers distributed over networks collaborate to control a secure session between the end-to-end peers. The Internet Engineering Task Force (IETF)’s direction is to use the horizontal model. One of the horizontal models introduced for the future 5G mobile networks is Software-Defined Networking (SDN) [10,11,12,13]. SDN is an emerging networking paradigm that changes the limitations of the existing vertical model including LTE network control model, which separates the network’s control plane from the data plane [14]. In SDN, the control plane is implemented in a logically centralized controller, simplifying the policy enforcement and network function configuration. The centralized network controller in the control plane manages the intelligence and state of the entire network for the network functions such as security management and mobility management [15,16]. However, the current network legacy devices are not yet ready to implement the horizontal model [17,18,19,20].

**Figure 2:**
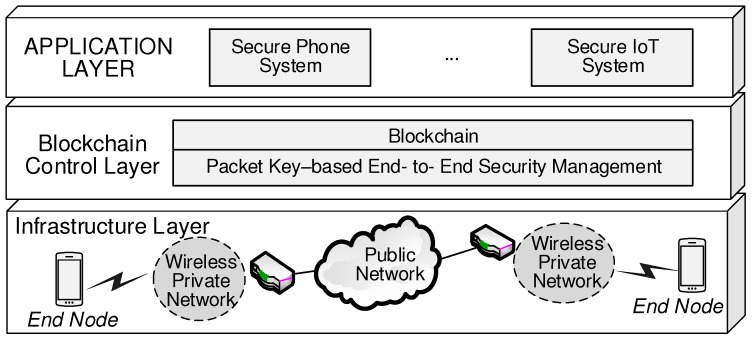
End-to-end packet key-based security management on the blockchain control plane.

The goal of this paper is to develop a blockchain-based security management (BSM) scheme which utilizes a packet key-based system to perform better and extends the scope in security management from the local area to the end-to-end security service range. As depicted in Figure 2, our BSM structure is close to the administrative control that the SDN-based horizontal model operates with. However, while the software-defined controller on the control plane functions as the control center, the BSM scheme utilizes one of the most innovative features of the blockchain where there is no central server running. BSM operates between two end-to-end peers without environments of the distributed servers and the centralized controller [21,22]. Thus, this idea gives significantly advantageous effects on the end-to-end security management by reducing the complexity of the system deployment and latency taken for the end-to-end secure session set up. From the latency viewpoints, our BSM scheme is more advantageous in security management because security parameter agreements between end-to-end peers can be easily obtained via blockchain. Furthermore, considering low latency of 1 ms in 5G networks (10 to 20 ms for 4G), our BSM scheme is more useful in 5G environments because it requires less computational load during secure session setup and the latency to the setup secure session is mainly affected by the network delay components [23,24,25].

The rest of this paper is organized as follows. In Section 2, this paper proposes and explains the BSM scheme. Then, Section 3 describes the improvement effects of the proposed BSM scheme. This paper will be concluded in Section 4.

## 2. Blockchain-Based Security Management (BSM) Scheme

### 2.1. Security Management on the Blockchain Control Plane

**Figure 3:**
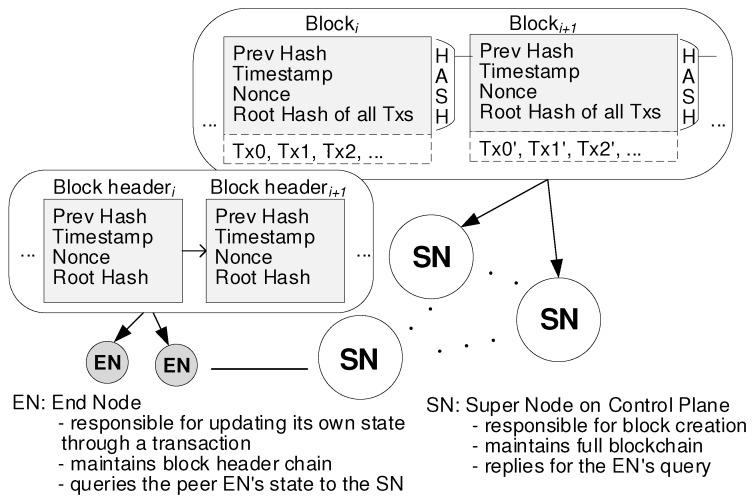
Blockchain-based Security Management (BSM) network architecture.

As shown in Figure 3, the blockchain-based security management (BSM) network architecture explains the feature of the control plane. The steps to run the network are as follows:

1. New transactions, which include ToALL Tx and Peer-fitted Tx, are sent to the nearest super node (SN). After the SN receives the transaction message, it broadcasts the message to all SNs. Each transaction message contains several pieces of field data for security management, which will be described later.
2. Like Bitcoin, our BSM scheme uses the consensus algorithm of PoW (Proof of Work). Thus, the PoW mining involves numerous hashing attempts. In the BSM network SNs are only able to add a new block to the blockchain if the distributed SNs reach consensus and agree that the block has provided by the miner is a valid proof of work. Differently from Bitcoin SNs are pre-determined at the stage of the system deployment. In addition, the BSM scheme limits the average block creation period within 1 second. The SN collects new transactions into a block and performs on finding a proof-of-work for its block. There are two kinds of blockchains: full blockchain and block-header chain. Here, the SN maintains the full blockchain while EN usually maintains the block-header chain. Later, when the end node (EN) needs a certain transactio information, it sends a query message to the nearest SN. Then, the SN searches the corresponding transaction data from the full blockchain and sends replies to the EN.
3. When an SN finds a proof-of-work, it broadcasts the block to all SNs.
4. SNs accept the block only if all transactions in it are valid.
5. Nodes imply their acceptance of the block by working on creating the next necessary block in the chain, using the hash of the accepted block as the previous hash. SNs and ENs will always keep working on extending full blockchains and block-header chains respectively.
6. The EN is responsible for updating its valid information for security management by pushing them into the blockchain. The valid information is contained in either ToALL Tx or Peer-fitted Tx.

Here, we define the latency from the moment a new transaction is announced to the network until the event that the transaction successfully gets in the blockchain as Time-to-Get-in-Blockchain ($T_{GinB}$). It is easy to adjust the average of TGinB value by controlling the block creation difficulty because our BSM scheme is based on the private blockchain among the SNs differently from the public blockchain where at least 51% miners have to agree for changing the difficulty [26]. Our BSM scheme adjusts this difficulty to target 1 second between blocks. The period of 1 second for $T_{GinB}$ means that it only takes 1 second on average for a certain EN’s new information for security management to become available to other ENs since the transaction information becomes valid on the blockchain after 1 second from the moment when the transaction was sent to the network.

### 2.2. BSM Wallet

The following information, which is maintained in the wallet, is used for the security management.

- Prime number (*q*) and primitive root (α): global parameters in the Diffie–Hellman key exchange.
- An array of secret values ($X_{EN}$s), that is, Secret Value Vector (SVV), for packet key agreements.

The tasks performed by the wallet software also include:

- generates the corresponding public key (pubKey) and the hash address (My_Hash_Addr),
- generates an array of blind keys ($Y_{EN}$s), that is, Blind Value Vector (BVV), using the equation of $Y_{EN}=\alpha^{X_{EN}}mod\;q$.
- generates an array of index values ($H_{EN}$s), that is, Blind Value Hash Table (BVHT), using the equation of $H_{EN}=$ Hash($Y_{EN}$). ‘Hash’ is a one-way hash function which accepts the blind value as input ($Y_{EN}$) and produces a fixed-size message digest as output ($H_{EN}$).

### 2.3. Security Parameter Agreement between Peers by Using Blockchain

**Figure 4:**
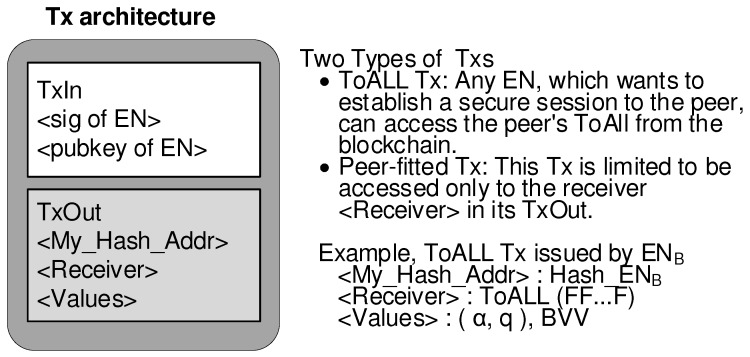
BSM transaction architecture.

Transactions, which grow without ceasing, are stored into a distributed database called the blockchain. There are two types of transactions: ToALL Tx and Peer-fitted Tx. Each EN sends its ToALL Tx to the network in advance. This process belongs to the registration step. Then, the ToALL Tx allows a certain session initiator to reach the ToALL transaction information of the session responder. The Peer-fitted Tx is sent to the network in order that the session responder obtains the session initiator’s Tx information during the session initiation stage. As illustrated in Figure 4, the BSM transaction consists of Transaction Input (TxIn) and Transaction Output (TxOut). The TxIn contains the signature and the public key computed from the EN’s private key which creates the transaction. The first field of TxOut contains the hash address that identifies the owner of this transaction.

**Figure 5:**
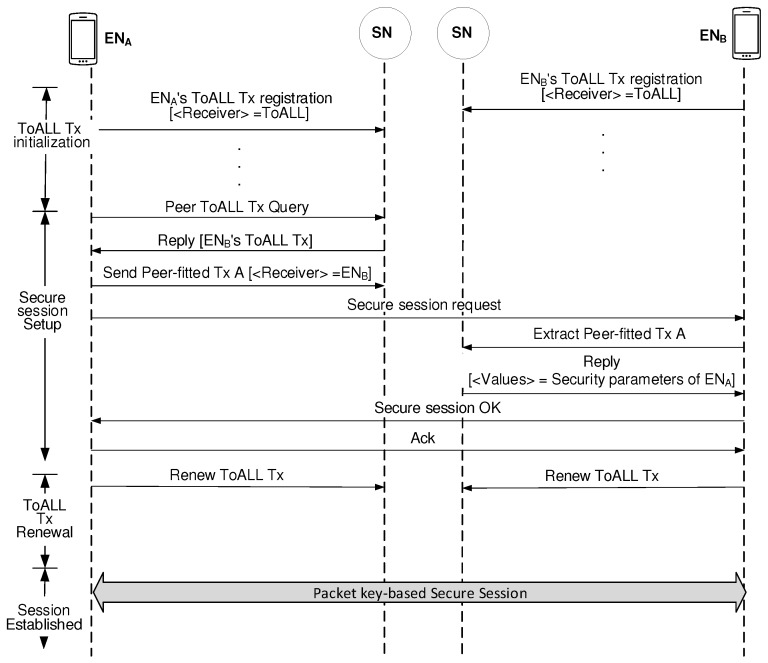
Security parameter agreement between peers by using blockchain.

Figure 5 illustrates how the SNs handles ToALL Txs and Peer-fitted Txs to establish the packet key-based secure session. Each EN, which needs the blockchain-based security management services, should store its ToALL Tx information into the blockchain in the pre-session stage. This process belongs to ToALL Tx initialization stage. Now, let’s assume the case that $EN_{A}$ wants to set up a packet key-based secure session with $EN_{B}$. $EN_{A}$ first uses the Query/Reply procedure to obtain the $EN_{B}$’s ToALL Tx information. After $EN_{A}$ succeeds to obtain $EN_{B}$’s ToALL Tx information, it sends the secure session request message destined to $EN_{B}$ along with sending the Peer-fitted Tx to the network. When $EN_{B}$ receives the secure session request message from $EN_{A}$, it extracts the Peer-fitted Tx from the blockchain. Then, all parameters for security management are agreed between the peers. Then, both peers renew their ToALL Txs. As a result of this renewal process, every session uses a different set of security parameters which contribute to guarantee a much more secure session.

### 2.4. Ever-Growing Series of Blocks and Blockchain Access

#### 2.4.1. Creation of ToALL Tx Blocks

**Figure 6:**
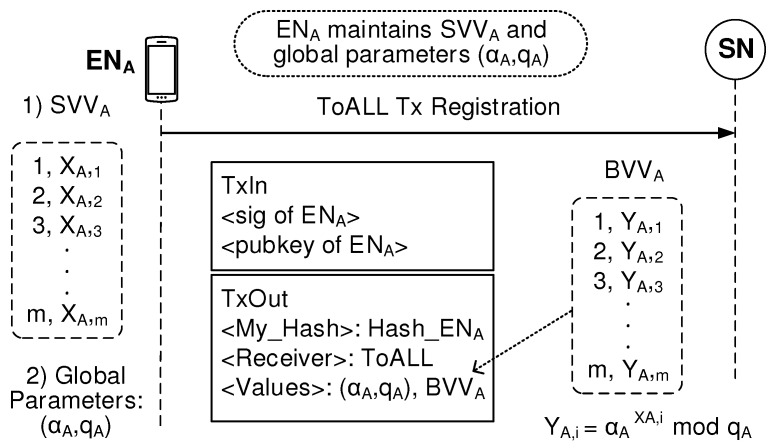
ToALL Tx registration.

The blockchain is a distributed database holding all the BSM transactions, that is, ToALL and Peer-fitted Txs, and keeps them secure. Figure 6 explains how the ToALL Tx information is stored in the blockchain. When a BSM application of $EN_{A}$ begins to work, $EN_{A}$ creates and maintains *m*-sized Secret Value Vector of $EN_{A}$, $SVV_{A}=\left[\left(1,X_{A,1}\right),\left(2,X_{A,2}\right),\left(3,X_{A,3}\right),\cdots ,\left(m,X_{A,m}\right)\right]$. Then, $EN_{A}$ calculates corresponding Blind Value Vector of $EN_{A}$, $BVV_{A}=[\left(1,Y_{A,1}\right),\left(2,Y_{A,2}\right),\left(3,Y_{A,3}\right),\cdots ,\left(m,Y_{A,m)}\right]$ where $Y_{A,i}=\alpha_{A}$${}^{X_{A,i}}$mod $q_{A}$, i=1,2,3,⋯,m. $EN_{A}$ sends ToALL Tx, which contains the value field of global parameters ($\alpha_{A},q_{A}$) and $BVV_{A}$, to the network expecting that those values are successfully stored in the blockchain within 1 second. Because of this registration process, the ToALL Tx information converts to public status so that any peer EN can be available to reach that ToALL Tx via the blockchain.

#### 2.4.2. Obtaining the Peer’s Information for Security Management

**Figure 7:**
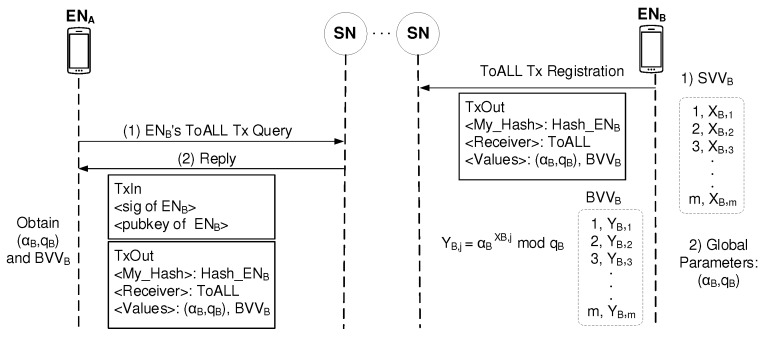
Tx information access by Query/Reply mechanism.

The procedure of a secure session establishment begins with the Query/Reply mechanism to obtain the peer’s information for security management. Figure 7 shows how $EN_{A}$ as a session initiator obtains the session responder $EN_{B}$’s information. $EN_{A}$ send Query for $EN_{B}$’s ToALL information to the nearest SN. Because $EN_{B}$ already registered its ToALL Tx to the blockchain, $EN_{A}$ can receive the reply of $EN_{B}$’s ToALL information from its nearest SN. Therefore, $EN_{A}$ can obtain <Values> in $EN_{B}$’s ToALL Tx: $\left(\alpha_{B},q_{B}\right)$ and $BVV_{B}$.

#### 2.4.3. Creation of Peer-Fitted Block

Because of the Query/Reply mechanism, $EN_{A}$ obtains $\left(\alpha_{B},q_{B}\right)$ and $BVV_{B}$. Here, for the scalability of our BSM scheme, it is assumed that the session initiator and the session responder use different global parameters. This means that our BSM scheme can be used to secure end-to-end peers which belong to different groups. If the BSM scheme operates between end-to-end peers in the same group, the use of the Peer-fitted Tx is not necessary. Because the BSM scheme is scalable, it requires the additional steps to handle the Peer-fitted Tx. The additional steps are as follows.

- The session initiator creates the Peer-fitted Tx.
- The session initiator sends the Peer-fitted Tx to the network.
- Some SN create a new block, which includes the Peer-fitted Tx and uses it to extend the blockchain.
- The session responder finally extracts the Peer-fitted Tx from the blockchain.

Those steps contribute to increase latency to complete the procedure of the secure session setup. This increased latency issue results in limitation of the BSM scheme.

**Figure 8:**
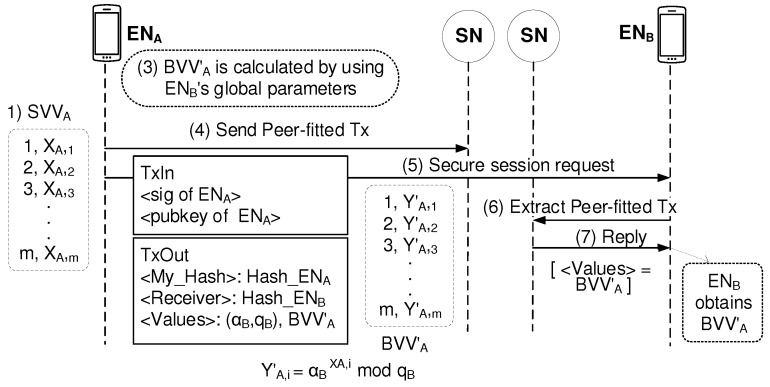
Sending Peer-fitted Blind Value Vector information via blockchain.

Then, using $\left(\alpha_{B},q_{B}\right)$, $EN_{A}$ calculates $BVV_{A}^{\prime }$ of $[\left(1,Y_{A,1}^{\prime }\right),\left(2,Y_{A,2}^{\prime }\right),\left(3,Y_{A,3}^{\prime }\right),\cdots ,\left(m,Y_{A,m)}^{\prime }\right]$ based on $SVV_{A}$ of $\left[\left(1,X_{A,1}\right),\left(2,X_{A,2}\right),\left(3,X_{A,3}\right),\cdots ,\left(m,X_{A,m}\right)\right]$. As shown in Figure 8, $EN_{A}$ sends Peer-fitted Tx to the network where the Tx contains $BVV_{A}^{\prime }$ where the component of $Y_{A,i}^{\prime }$ is calculated by using the equation of ${\alpha_{B}}^{X_{A,i}}$ mod $q_{B}$. When (5) Secure session request arrives at $EN_{B}$, $EN_{B}$ can obtain $BVV_{A}^{\prime }$ via (6) Extract Peer-fitted Tx and (7) Reply.

#### 2.4.4. Security Parameter Agreements between Peers

**Figure 9:**
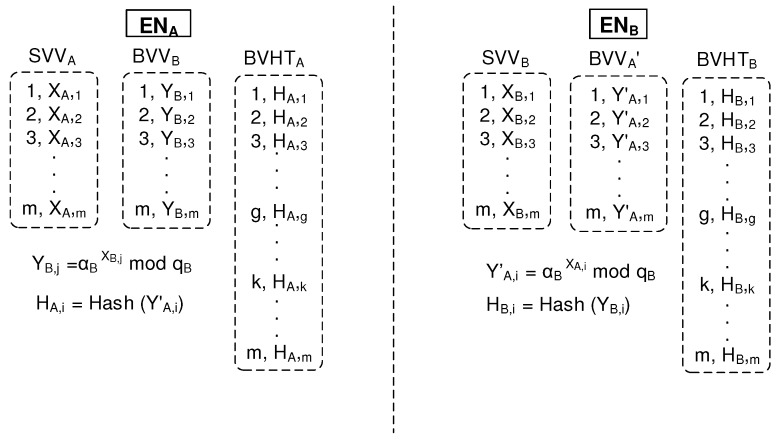
Security parameter agreements between peers.

As shown in Figure 9, $EN_{B}$ can maintain security-related parameters: $SVV_{B}$, $BVV_{A}^{\prime }$, and the index table of $BVHT_{B}$. Here, $H_{B,i}$, which is the hashed value of $Y_{B,i}$, can be used as the index of security parameters selected. In addition, $EN_{A}$ side can use security-related parameters: $SVV_{A}$, $BVV_{B}$, and the index table of $BVHT_{A}$. Now, both sides are ready to use the packet-key system.

#### 2.4.5. Renewal Process for the Security Parameters

**Figure 10:**
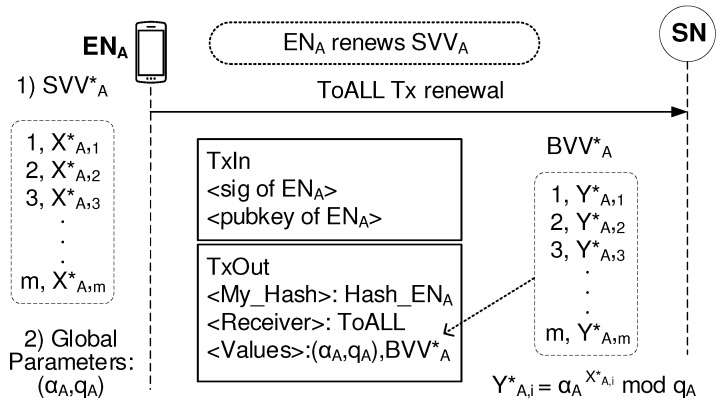
ToAll Tx renewal.

The secure aspect of our BSM system is due to the fact that each secure session establishment procedure uses the different set of security parameters: SVV, BVV, and BVHT. Each EN uses a different SVV of $\left[\left(1,X_{1}\right),\left(2,X_{2}\right),\left(3,X_{3}\right),\cdots ,\left(m,X_{m}\right)\right]$ every session. This means that once SVV is used, the previous SVV is replaced with new $SVV^{*}$. Thus, the $BVV^{*}$, which calculated from the new $SVV^{*}$, needs to be made public. This is the ToALL Tx renewal process shown in Figure 10. $EN_{A}$ creates $SVV_{A}^{*}$ of $\left[\left(1,X_{A,1}^{*}\right),\left(2,X_{A,2}^{*}\right),\left(3,X_{A,3}^{*}\right),\cdots ,\left(m,X_{A,m}^{*}\right)\right]$ and calculates the corresponding $BVV_{A}^{*}$ of $[\left(1,Y_{A,1}^{*}\right),\left(2,Y_{A,2}^{*}\right),\left(3,Y_{A,3}^{*}\right),\cdots ,\left(m,Y_{A,m)}^{*}\right]$ using the equation of $Y_{A,i}^{*}=\alpha_{A}$ ${}^{X_{A,i}^{*}}$ mod $q_{A}$. Then, the ToALL Tx, which includes the value field of $\left(\alpha_{A},q_{A}\right)$ and $BVV_{A}^{*}$, is sent to the network.

### 2.5. Operation of the Packet Key Bbased Secure Data Session

**Figure 11:**
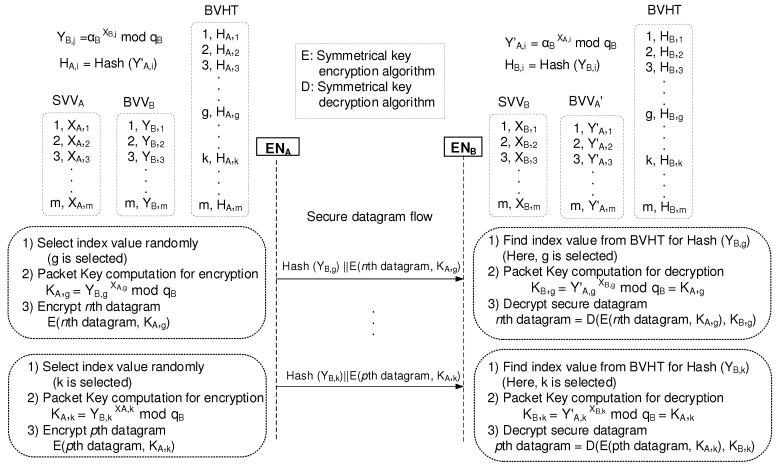
Packet key encryption and decryption for the secure datagram flow.

Figure 11 explains how the proposed scheme encrypts the whole session by applying a different packet key to each datagram under the condition that $EN_{A}$ and $EN_{B}$ maintain $\left[\left(1,X_{A,1}\right),\left(2,X_{A,2}\right),\left(3,X_{A,3}\right),\cdots ,\left(m,X_{A,m}\right)\right]$ and $\left[\left(1,X_{B,1}\right),\left(2,X_{B,2}\right),\left(3,X_{B,3}\right),...\left(m,X_{B,m}\right)\right]$, respectively. In addition, $EN_{A}$ and $EN_{B}$ are ready to use $[\left(1,Y_{B,1}\right),\left(2,Y_{B,2}\right),\left(3,Y_{B,3}\right),\cdots ,\left(m,Y_{B,m)}\right]$ and $[\left(1,Y_{A,1}^{\prime }\right),\left(2,Y_{A,2}^{\prime }\right),\left(3,Y_{A,3}^{\prime }\right),\ldots \left(m,Y_{A,m)}^{\prime }\right]$, respectively. Then, when $EN_{A}$ starts to send *n*th datagram to $EN_{B}$, $EN_{A}$ selects an index value randomly. Here, we assume that *g* is selected. Then, $EN_{A}$ computes the packet key of $K_{A,g}$ using the equation of ${Y_{B,g}}^{X_{A,g}}\;mod\;q_{B}$. Now, $EN_{A}$ can encrypt the *n*th datagram using the resultant packet key which results in E (*n*th datagram, $K_{A,g}$), where E is any symmetrical key encryption algorithm. Finally, $EN_{A}$ sends the encrypted *n*th datagram together with the index value of *g*. When $EN_{B}$ receives the encrypted *n*th datagram from $EN_{A}$, $EN_{B}$ finds the index value of *g*. Then, $EN_{B}$ computes the packet key of $K_{B,g}$ using the equation of ${Y_{A,g}^{\prime }}^{X_{B,g}}\;mod\;q_{B}$. Now, $EN_{B}$ can decrypt the encrypted *n*th datagram using the resultant packet key, which results in D(E(*n*th datagram, $K_{A,g}$), $K_{B,g}$), where D is the same symmetrical key Decryption algorithm as $EN_{A}$. Finally, $EN_{B}$ obtains the *n*th datagram.

## 3. Improvement Effects of the Proposed BSM Scheme

### 3.1. Effects on Security

The most attractive feature of the packet key scheme is that there is little chance to reuse the same packet key for a continuous packet data stream. Additional advantageous effects are as follows. As shown in Figure 10, the renewal process of the BSM scheme enables the different packet data session to use completely different security parameters for security management. Therefore, our BSM scheme is secure against the powerful eavesdroppers who use brute-force approaches even to try after-transmission attacks. Our BSM scheme also uses BVHT to indicate the index value associated with the packet key used. The one-way hash function accepts the blind value selected from the BVV as input and produces a fixed-size hash value as output, which is used as the index value. As shown in Figure 11, each datagram in a secure datagram flow contains the corresponding index value for a certain blind value. This means that our BSM scheme protects the blind values while the existing Diffie–Hellman key exchange method exposes the blind values. Instead of the blind values, exposing the hash values to the possible attackers contributes to our BSM scheme to lead to very secure end-to-end data transfer systems against active attacks such as falsification of data and transactions.

### 3.2. Effects on Latency

**Figure 12:**
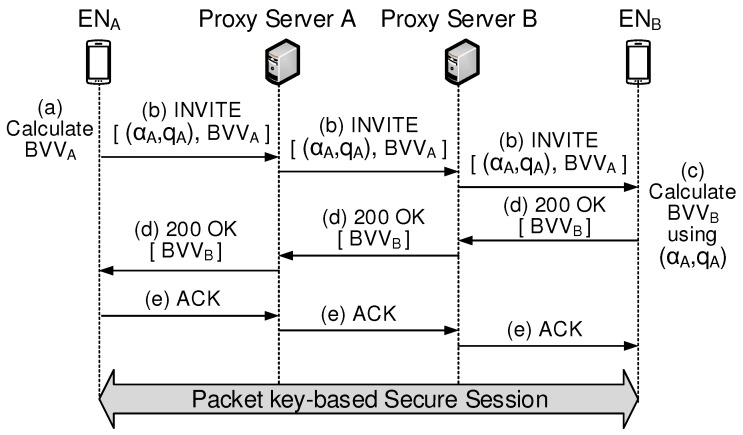
Secure Session Initiation Protocol (SIP) model for the packet key-based encrypted data flow.

In SIP-based VoIP call operation, an end user sends SIP requests to initiate a session. Figure 12 shows a series of steps required to complete a packet key-based secure VoIP session set up between two ENs.

This paper calls the existing SIP plus packet key model shown in Figure 12 a “vertical model”. Some assumptions are necessary to perform the comparative analysis with respect to total latency to complete security management between $EN_{A}$ and $EN_{B}$, where they are located in different domains. This paper assumes three types of delays, that is (1) $T_{Intra}$: intra-domain delay caused in intra-domain links, (2) $T_{E2E}$: end-to-end delay caused in end-to-end path, (3) $T_{BVV}$: processing delay caused to compute BVV, and (4) $T_{GinB}$: latency for creating a block.

**Figure 13:**
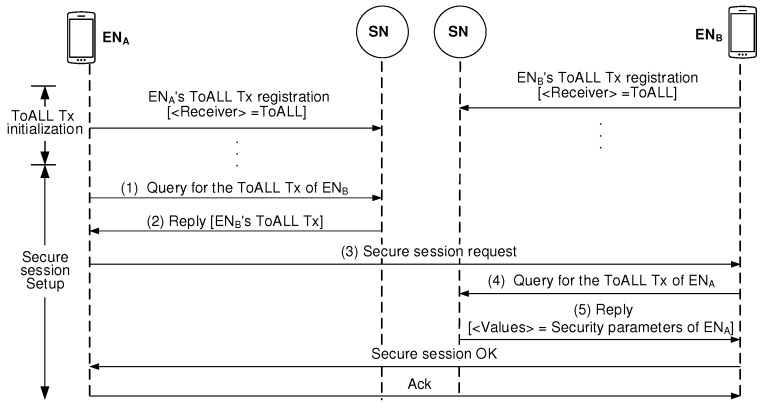
Secure session setup procedure in the non-scalable BSM model.

Table 1 shows comparisons on latency needed to set up a packet key-based secure session between the proposed BSM model, the non-scalable BSM model, and the vertical model. Figure 13 shows the secure session setup procedure for the non-scalable BSM scheme. Compared to the proposed BSM scheme in Figure 5, it is shown that the secure session setup procedure for the non-scalable BSM scheme is much simpler.

The latency of $T_{BVV}$ changes depending on the size of BVV as well as the size of the secret value. Here, the size of the secret value, as well as the blind value, is the same as that of the packet key. Recall that calculating each component of the BVV needs the corresponding latency for the computation of ${\alpha_{A}}^{X_{A,i}}\;mod\;q_{A}$. Table 2 shows experimental data on computational latency needed to compute each blind value for a given secret value. The results were obtained from the testbed with 2.2GHz i7-8750H CPU. Then, the *m*-sized BVV requires *m* times the above latency.

**Figure 14:**
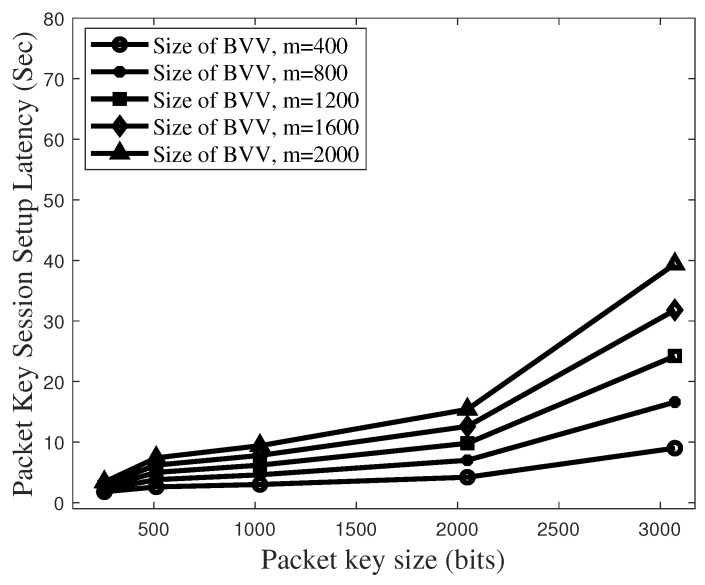
Total latency to set up a packet key-based secure session in the BSM model.

Figure 14 plots the total latency to set up the packet key-based secure session in the BSM model, which corresponds to the latency of $2T_{Intra}+1T_{BVV}+1T_{GinB}$. Here, we assume that 2 × $T_{Intra}$ is 0.4 s and $T_{GinB}$ is one second.

**Figure 15:**
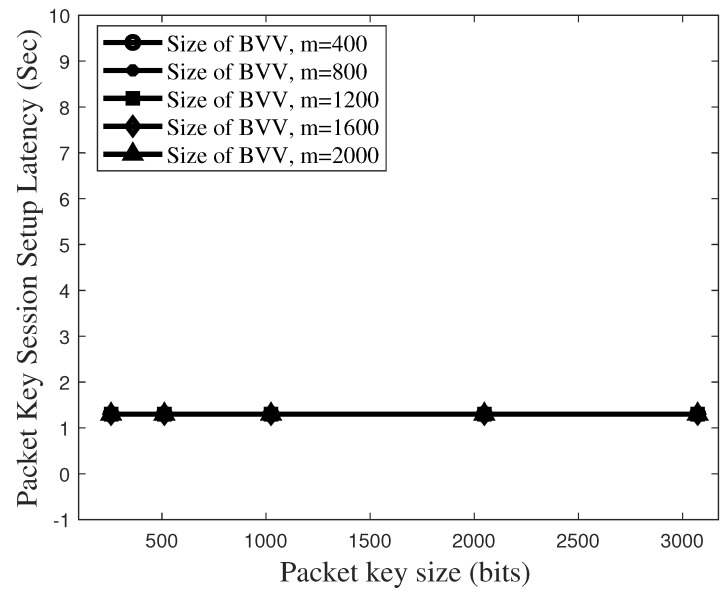
Total latency to set up a packet key-based secure session in the non-scalable BSM model.

Figure 15 plots the total latency to set up the packet key-based secure session in the non-scalable BSM model, which corresponds the latency of $4T_{Intra}+1T_{E2E}$. Here, we assume that 4 × $T_{Intra}$ is 0.8 s and $T_{E2E}$ is 0.5 s. For the non-scalable BSM model, the total latency is independent of the parameter of $T_{BVV}$. It is shown that the total latency to setup the packet key-based secure session can be dramatically reduced if the proposed BSM scheme operates in the limited scale.

**Figure 16:**
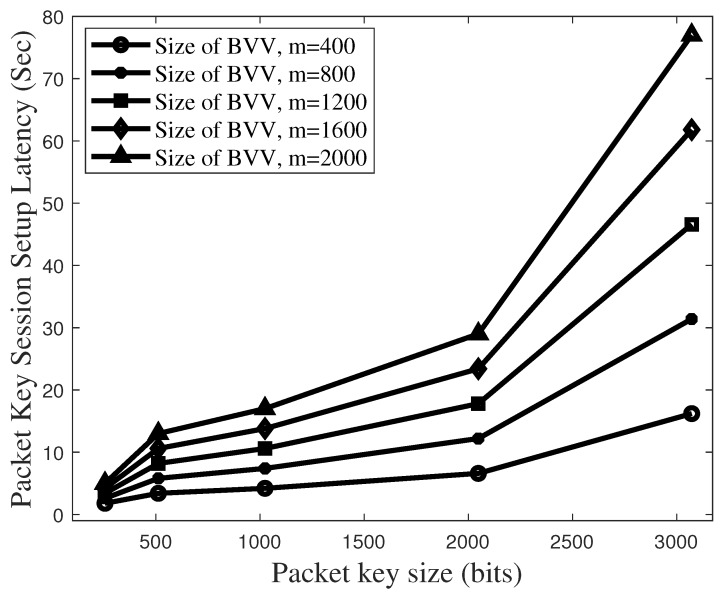
Total latency to set up a packet key-based secure session in the vertical model.

Figure 16 plots the total latency to set up the packet key-based secure session in the vertical model, which corresponds to the latency of $2T_{E2E}+2T_{BVV}$. Here, we assume that 2 × $T_{E2E}$ is one second.

The use of packet key guarantees very secure data flow [27]. However, the latency to set up the secure session needs to be limited to below several seconds. In Figure 14, it is shown that the BVV size of 1600 and the key size of 2048 bits are enough to satisfy the session set up latency below 10 s. On the other hand, according to Figure 16, the condition of 1600-sized BVV and 2048-sized key causes the latency to rise beyond 20 s. If the vertical model uses the BVV size of 2000 and the key size of 2048 bits, its packet key session setup latency tends to increase up to 30 s. As a result, it is proved that, compared with the vertical model, our BSM scheme effects are better by almost 200% on latency to set up a secure session while it provides the same security level as the vertical model.

Table 3 shows comparisons on two kinds of network delay components for the 3G, 4G and 5G networks. According to [24], $T_{Intra}$ can be reduced to 2 ms in 5G networks while $T_{Intra}$ of 20 ms is required in 4G networks. Then, it is reasonable to consider that $T_{E2E}$ values in 4G networks and 5G networks are 50 ms and 5 ms, respectively.

**Figure 17:**
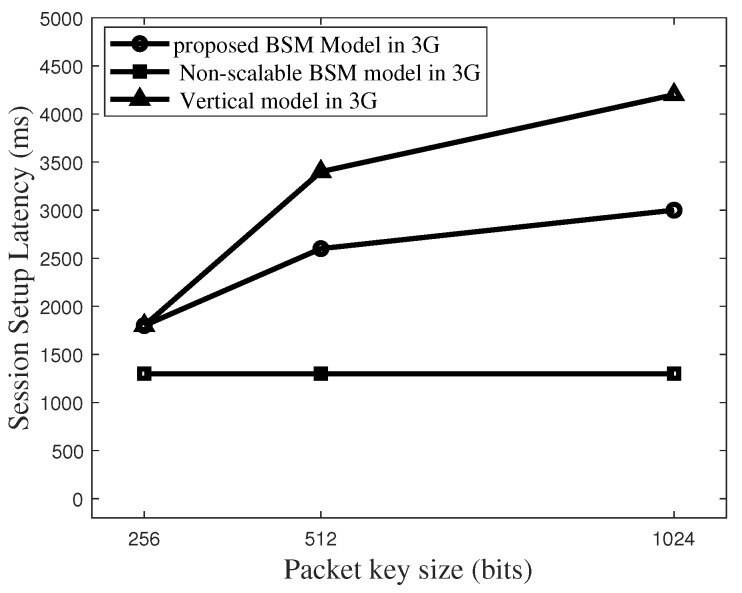
Total latency to set up a packet key-based secure session in 3G networks for the case size of Blind Value Vector (m) = 400.

Figure 17 plots the latency comparisons for different models. Those latency results are based on the conditions that 3G networks work and the size of BVV is 400, that is, m = 400. Considering that in the packet key scheme different keys are supplied for different packets, packet key size of around 512 bits is enough to satisfy the secure session. It is shown that either the proposed BSM model or the non-scalable BSM model guarantees latency level of 2500 ms even in 3G networks.

**Figure 18:**
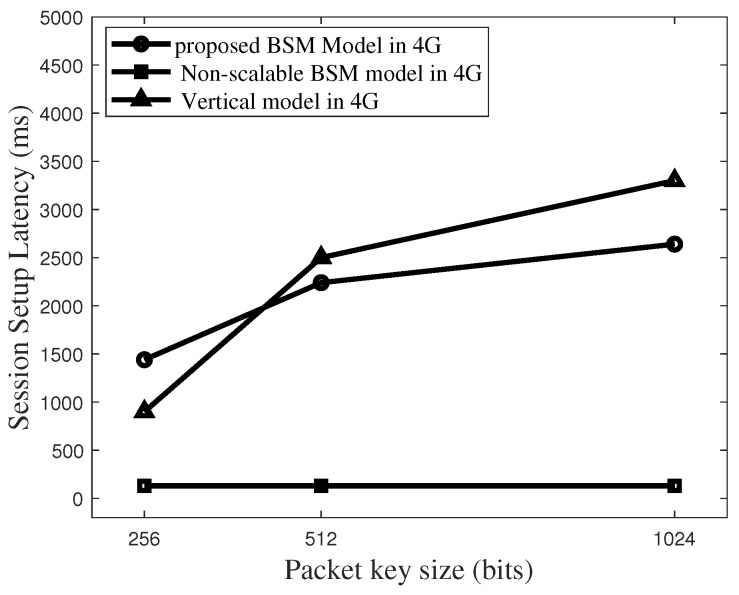
Total latency to set up a packet key-based secure session in 4G networks for the case size of Blind Value Vector (m) = 400.

Figure 18 plots the latency comparisons for different models in 4G environments where m = 400. It is shown that the key size of 512 bits is the turning point where the proposed BSM scheme performs better on latency compared with the vertical model. In addition, the non-scalable BSM model guarantees very low latency level as the 4G networks guarantee fast data rates.

**Figure 19:**
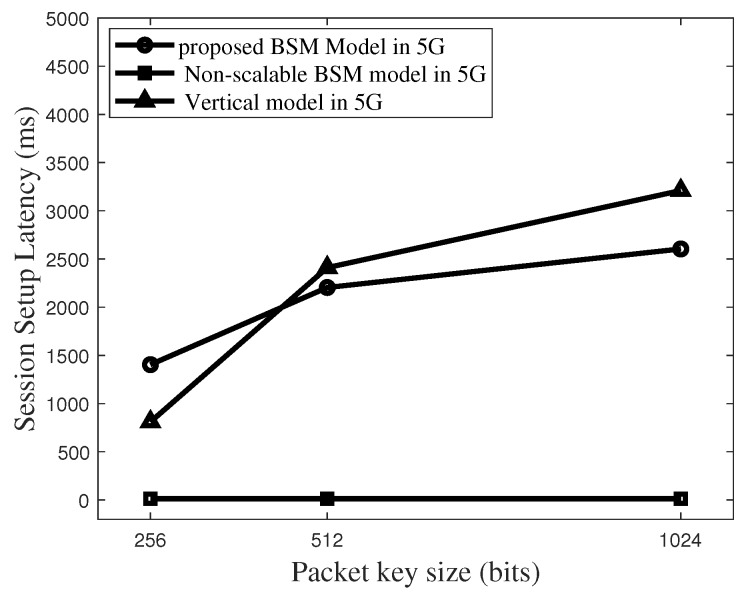
Total latency to set up a packet key-based secure session in 5G networks for the case size of Blind Value Vector (m) = 400.

Figure 19 plots the latency comparisons for different models in 5G environments where m = 400. In 5G environments, with the key size of 512 bits, latency level can be reduce to 2000 ms in the BSM model. In addition, the non-scalable BSM model guarantees much less latency level as the 5G networks serve very fast data rates.

## 4. Conclusions

Existing phone systems, such as SIP-based VoIP call systems, use the vertical model to solve issues relating to the security management. As of now, as IETF suggests, an alternative solution is to use the SDN horizontal model where a centralized software-defined network controller on the control plane is in charge of security management. The goal of this paper is to introduce blockchain technologies to manage a packet key-based security system, which can overcome the limitation that the vertical model is confronted with. While the packet key-based security system can provide very strong security strength, it needs a high computational power to agree on security parameters between peers at the packet key session setup phase. Our BSM scheme is advantageous from the perspective that, using blockchain, each peer can easily obtain the necessary parameters required to handle security management.

Advantageous effects in our BSM scheme result from the renewal process, which enables the different packet data sessions to use completely different security parameters for the security management. Therefore, our BSM scheme is secure against the powerful eavesdroppers who use brute-force approaches even to try after-transmission attacks. In addition, in our BSM scheme, corresponding hash values travel together with the secured datagrams so that even blind values cannot be exposed to the possible attackers. This contributes our BSM scheme to lead to very secure end-to-end data transfer systems against active attacks such as falsification of data and transactions. From the viewpoints of latency, the BSM scheme performs better by around 200% than the existing vertical model.

## Figures and Tables

**Table 1:** Comparisons on latency needed to set up packet key-based session.

Models	Latency to Setup a Secure Session
Proposed BSM model	2$T_{Intra}$ ((1), (2) in Figure 7)
	1$T_{BVV}$ ((3) in Figure 8)
	1$T_{GinB}$ ((4)+(5)+(6)+(7) in Figure 8)
	($2T_{Intra}+1T_{BVV}+1T_{GinB}$)
Non-scalable BSM model	2$T_{Intra}$ ((1), (2) in Figure 13)
	1$T_{E2E}$ ((3) in Figure 13)
	2$T_{Intra}$ ((4), (5) in Figure 13)
	($4T_{Intra}$ + 1$T_{E2E}$)
Vertical model	2$T_{E2E}$: ((b), (d) in Figure 12)
	2$T_{BVV}$ ((a), (c) in Figure 12)
	($2T_{E2E}+2T_{BVV}$)

**Table 2:** Key-size varying latency for computing a blind value.

Packet Key Size (bits)	Latency to Compute ${\alpha_{A}}^{X_{A,i}}\;\mathrm{mod}\;q_{A}$ (ms)
256	1
512	3
1024	4
2048	7
3072	19

**Table 3:** Network delays for 3G, 4G, and 5G networks.

Networks	$T_{\mathrm{Intra}}$	$T_{E2E}$
3G	200 ms	500 ms
4G	20 ms	50 ms
5G	2 ms	5 ms
